# β-Sitosterol Enhances the Anticancer Efficacy of Oxaliplatin in COLO-205 Cells via Apoptosis and Suppression of VEGF-A, NF-κB-p65, and β-Catenin

**DOI:** 10.3390/ijms262210897

**Published:** 2025-11-10

**Authors:** Sahar Khateeb, Fahad M. Almutairi, Adel I. Alalawy, Amnah Obidan, Mody Albalawi, Rehab Al-Massabi, Hanan Abdulrahman Sagini, Samah S. Abuzahrah, Eman F. S. Taha

**Affiliations:** 1 Department of Biochemistry, Faculty of Science, University of Tabuk, Tabuk 71491, Saudi Arabia; 2 Department of Biological Sciences, College of Sciences, University of Jeddah, Jeddah 21959, Saudi Arabia; 3 Health Radiation Research Department, National Center for Radiation Research and Technology, Egyptian Atomic Energy Authority (EAEA), Cairo 11787, Egypt

**Keywords:** colon cancer, β-Sitosterol, oxaliplatin, apoptosis, cell cycle, MTT, *Bax/Bcl2* ratio

## Abstract

Colon cancer (CC) is a common malignancy characterized by poor prognostic outcomes and considerable mortality. Oxaliplatin (OXP) is commonly used in the treatment of CC; however, its efficacy may be limited by side effects and the development of resistance. β-sitosterol (β-Sit), a phytosterol derived from plants, has been documented to be effective in the treatment of tumors. This study aimed to investigate the potential of β-Sit to enhance the antitumor efficacy of OXP in COLO-205 cells, focusing on apoptosis induction and suppression of the vascular endothelial growth factor A (VEGF-A)/survival pathway. Molecular docking studies were performed to assess the binding affinity of β-Sit with the target proteins B-cell lymphoma 2 (Bcl-2), phosphoinositide 3-kinase (PI3K), and VEGF receptor-2 (VEGFR-2). COLO-205 cells were treated with OXP, β-Sit, or a combination of OXP + β-Sit for 48 h. The combination treatment substantially lowered the IC_50_ achieved with 3.24 µM of OXP and 36.01 µM of β-Sit, compared to 25.64 µM for OXP alone and 275.9 µM for β-Sit alone, demonstrating a pronounced synergistic impact. The combined therapy altered the cell cycle distribution by decreasing the number of cells in the G0/G, S, and G2/M phases, coupled with an increase in the Sub-G1 population. Furthermore, apoptosis was augmented by a shift in cell death from necrosis to late apoptosis, as indicated by an increased *BAX/BCL2* ratio relative to each treatment alone. Moreover, the inhibitory effect on angiogenesis was enhanced via the reduction of VEGF-A, and β-catenin and nuclear factor κB (NF-κB-p65) were suppressed, thereby preventing the growth and survival of resistant cancer cells. Additionally, molecular docking supported high binding affinities of β-Sit to Bcl-2, PI3K, and VEGFR-2. This study highlights the potential of β-Sit to enhance the anti-cancer efficacy of OXP in CC.

## 1. Introduction

Colon cancer (CC) is a prevalent malignancy characterized by poor prognostic outcomes, high rates of disease recurrence, and significant mortality. CC impacted approximately 1.9 million individuals worldwide and accounted for almost 0.9 million premature fatalities in 2020, ranking as the second most fatal malignancy [1]. Radiotherapy and chemotherapy are the primary treatment modalities for resectable and advanced CC [2,3]. Nonetheless, existing clinical therapies exhibit limitations, including the severe adverse effects associated with chemotherapy agents and the development of tolerance with prolonged administration [4,5].

Platinum-based drugs, such as oxaliplatin (OXP), are employed to treat various cancers; nevertheless, their effectiveness is often constrained by toxicities [6] and the development of resistance [7]. Mechanisms of OXP resistance in malignancies encompass modifications in DNA damage responses, cell death pathways, nuclear factor κB (NF-kB) signaling, and cellular transport [8]. Despite the OXP-resistant cancer cells, several studies indicate that chemo-resistant subpopulations may be more susceptible to adjuvant therapies [8,9,10,11]. These limitations highlight the requirement for combinatorial strategies employing natural compounds with traditional drugs such as OXP to enhance therapeutic efficacy and minimize adverse effects.

The intake of specific phytochemicals has been linked to a diminished risk of cancer, and their intrinsic safety and cost-efficiency make these dietary drugs a desirable choice for extensive and prolonged application in cancer chemoprevention [12]. β-Sitosterol (β-Sit) is the predominant phytosterol found in nearly all plant-based foods and several traditional Chinese herbs. It has been utilized in the treatment of various diseases due to its extensive biological functions, including anti-inflammatory [13], analgesic [14], and antidiabetic [15] properties, as well as its notable anticancer potential [16]. Previous studies have demonstrated that β-Sit promotes apoptosis in ovarian cancer cells [17] and triggers apoptosis in A549 and NCIH460 cell lines [18]. Furthermore, it has exhibited effectiveness in controlling the growth of malignant neoplasms, such as colorectal carcinoma and cholangiocarcinoma [19,20]. These findings suggest that the combination of β-Sit and OXP may augment anticancer effects through additive or synergistic interactions.

Although COLO-205 cells are commonly used in CC research, investigations of the impact of β-Sit on these cells are limited, particularly for signaling pathways including vascular endothelial growth factor A (VEGF-A), phosphoinositide 3-kinase (PI3K), mammalian target of rapamycin (mTOR), NF-κB-p65, and β-catenin. The potential synergistic effects of combining OXP with β-Sit on COLO-205 cells remain unexplored. Addressing these deficiencies may provide new insight into combinatorial therapeutic strategies for CC and improve the efficacy of chemotherapy. The study aimed to investigate the potential of β-Sit to enhance the antitumor efficacy of OXP in COLO-205 cells, focusing on apoptosis induction, suppression of VEGF-A-mediated angiogenesis, NF-κB-p65, and β-catenin.

## 2. Results and Discussion

### 2.1. Molecular Docking Results

**Figure 1:**
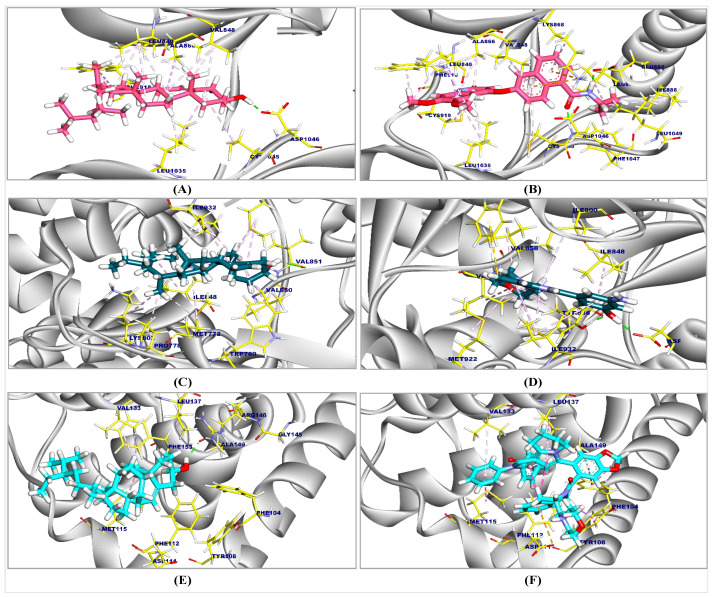
3D figure of the proposed binding mode of β-Sit against VEGFR-2, PI3K, and BCL2: (**A**) β-Sit against VEGFR-2, (**B**) Co-crystallized ligand complexed with VEGFR-2, (**C**) β-Sit against PI3K, (**D**) Co-crystallized ligand complexed with PI3K, (**E**) β-Sit against BCL2, and (**F**) Co-crystallized ligand complexed with BCL2.

β-Sit had promising affinity for vascular endothelial growth factor receptor-2 (VEGFR-2), PI3K, and Bcl-2, as indicated in Table 1. The proposed binding mode of β-Sit demonstrated a binding affinity of −7.45 kcal/mol against VEGFR-2. β-Sit showed thirteen hydrophobic π-alkyl interactions with Phe918, Leu840, Leu1035, Ala866, Cys1045, and Val848. Moreover, it interacted with Asp1046 by a hydrogen bond (H-bond) with a distance of 1.71 Å (Figure 1A). While the co-crystallized ligand (887) complexed with VEGFR-2 produced an affinity score of −8.13 kcal/mol and established 17 hydrophobic π-alkyl, π-sigma, and π-cation interactions with Phe1047, Leu889, Ile888, Leu1049, Lys868, Ala866, Leu1035, Phe918, Leu840, Val848, and Cys1045. Moreover, three H-bonds were noted with Cys919, Glu885, and Asp1046 with distances of 1.96, 1.79, and 1.87 Å (Figure 1B).

The binding mode of β-Sit to PI3K demonstrated a binding affinity of −7.98 kcal/mol. It established thirteen hydrophobic π-alkyl contacts with Ile848, Met922, Trp780, Val850, Ile932, Met772, Pro778, and Lys802. Additionally, it formed two H-bonds with Val851, with distances of 2.19 and 2.06 Å (Figure 1C). The co-crystallized ligand (84R) demonstrated an affinity score of −8.02 kcal/mol, interacting with Val850, Val851, Met922, Tyr836, Ile848, Ile932, Ile800, and Trp780 through eleven hydrophobic π-interactions. It also formed three H-bonds with Asp810, Tyr836, and Val851, with distances of 1.70, 1.74, and 1.68 Å, respectively (Figure 1D).

The binding mode of β-Sit demonstrated an affinity score of −6.88 kcal/mol against BCL2. It formed four hydrophobic π-alkyl connections with the side chains of Phe153, Val133, and Met115 amino acids. An H-bond was also detected with Ala149, facilitating interactions at a distance of 2.11 Å (Figure 1E). The co-crystallized ligand (F3Q), which inhibited BCL2, demonstrated an affinity score of −7.11 kcal/mol and established twelve hydrophobic interactions, including π-π, π-sigma, π-anion, π-alkyl, and H-bonds with Met115, Val133, Phe104, Leu137, Tyr108, Phe112, Asp111, Gly44, and Ala149, at a distance of 1.74 Å (Figure 1F).

### 2.2. Cytotoxic Evaluation

**Figure 2:**
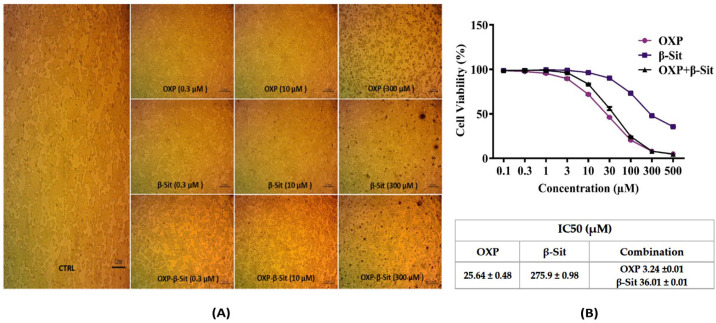
MTT cytotoxicity analysis on COLO-205 cells: (**A**) Depictive images of the MTT assay showing the effects of OXP, β-Sit, and their combination on cell viability at concentrations of 0.3, 10, and 300 µM. Scale bar: 0.2 mm. (**B**) The IC_50_ values for each treatment at various concentrations (0.1, 0.3, 1, 3, 10, 30, 100, 300, and 500 µM). Data represent the mean ± SD (*n* = 3). Control (CTRL), Oxaliplatin (OXP), β-sitosterol (β-Sit).

The 3-(4,5-dimethylthiazol-2-yl)-2,5-diphenyltetrazolium bromide (MTT) is one of the most frequently employed methods for assessing drug toxicity [21]. The cytotoxic effects of OXP, β-Sit, and their combination (OXP + β-Sit) on COLO-205 cells were determined using the MTT assay during a 48 h period to assess inhibitory concentration (IC50) value, as displayed in Figure 2A,B. The MTT assay images (Figure 2A) demonstrated a concentration-dependent effect on cell viability; however, OXP and β-Sit individually caused cytotoxicity at high doses. Their combination (OXP + β-Sit) produced markedly increased toxicity even at lower concentrations. Figure 2B displays the IC_50_ values for various treatments: OXP demonstrated an IC_50_ of 25.64 µM, β-Sit exhibited 275.9 µM, and the combination of OXP and β-Sit resulted in an IC_50_ achieved with 3.24 µM of OXP and 36.01 µM of β-Sit. This indicates a synergistic effect, enabling lower dosages of each compound while maintaining substantial cytotoxic efficacy against cancer cells, thereby supporting the study’s hypothesis regarding the effectiveness of combination therapy as a potent treatment strategy.

### 2.3. Effect of OXP, β-Sit, or Their Combination on Cell Cycle Distribution

**Figure 3:**
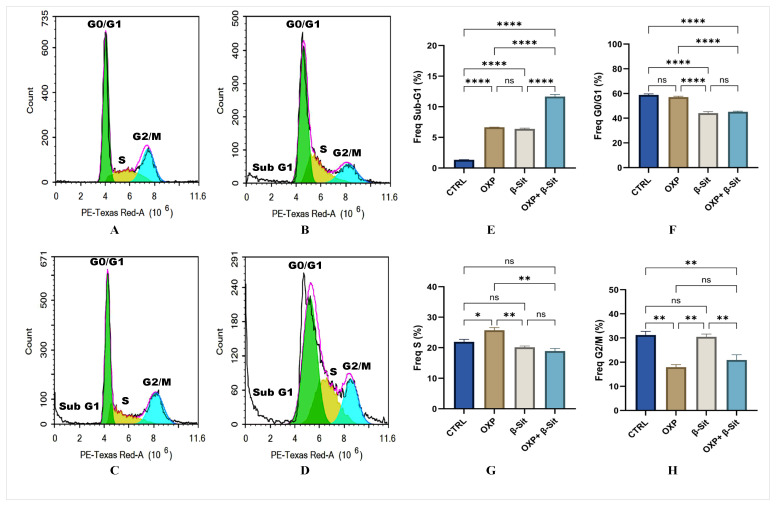
(**A**–**D**) Representative histograms of cell cycle analysis for COLO-205 cells following treatment at IC_50_ concentrations: (**A**) CTRL, (**B**) OXP, (**C**) β-Sit, and (**D**) OXP + β-Sit. (**E**–**H**) Quantitative analysis of cell cycle: (**E**) sub G1, (**F**) G0/G1, (**G**) S, and (**H**) G2/M phases. Data are presented as mean ± SEM (*n* = 3). The statistical analysis was conducted using ANOVA/Tukey’s post hoc test. ns = not significant, * *p* < 0.05, ** *p* < 0.01, **** *p* < 0.0001.

Flow cytometry was utilized to assess the specific stage of the cell cycle at which the cells were arrested [22]. Figure 3A–D displays the impact on the Sub-G1, G0/G1, S, and G2M phases following a 48 h treatment with OXP, β-Sit, or their combination (OXP + β-Sit). Compared to the CTR, treatment with OXP or β-Sit, either alone or in combination, significantly increased the number of cells in the Sub-G1 phase (*p* < 0.0001). Moreover, the combination therapy (OXP + β-Sit) caused a higher percentage of cells in the Sub-G1 phase compared to OXP or β-Sit (*p* < 0.0001) (Figure 3E), indicating enhanced apoptotic activity. This finding suggests that β-Sit enhances the cytotoxic efficacy of OXP by promoting apoptosis. Regarding the G0/G1 phase, treatment with OXP alone did not significantly reduce the proportion of cells in the G0/G1 phase; however, β-Sit, either alone or combined with OXP, results in a significant decrease in the number of cells in the G0/G1 phase (*p* < 0.0001) compared to the CTR. Furthermore, the combination treatment considerably reduced cell counts in comparison to OXP (*p* < 0.0001), with no notable change observed when compared to β-Sit alone (Figure 3F). This reduction indicates that β-Sit expedites the transition of cells from G0/G1 to apoptosis, thereby enhancing the cytotoxic efficacy of OXP in the combination therapy.

Furthermore, OXP treatment markedly increased cell accumulation in the S phase, indicative of S-phase arrest, in comparison to CTRL and other treatments. These data demonstrate that OXP induces cytotoxicity by causing S-phase arrest, indicative of its DNA-damaging properties. While β-Sit or the combination showed no significant alteration in S-phase distribution (Figure 3G). Moreover, during the G2/M phase, OXP, whether administered alone or in combination with β-Sit, significantly diminished cell numbers in the G2/M phase relative to the CTRL and β-Sit alone (*p* < 0.01) (Figure 3H). β-Sit maintained a substantial fraction of cells in G2/M (partial G2/M arrest), indicating distinct checkpoint regulation. Overall, the combination treatment exhibited the most notable effect on inhibiting cell cycle progression, where β-Sit enhances OXP efficacy by directing cells toward apoptosis (Sub-G1). These observations align with the prior study by Choi et al. [23], demonstrating that β-Sit can affect cell death pathways by increasing the sub-G1 cell population.

### 2.4. Assessment of Lymphocyte Forward and Side Scatter (FSC and SSC)

**Figure 4:**
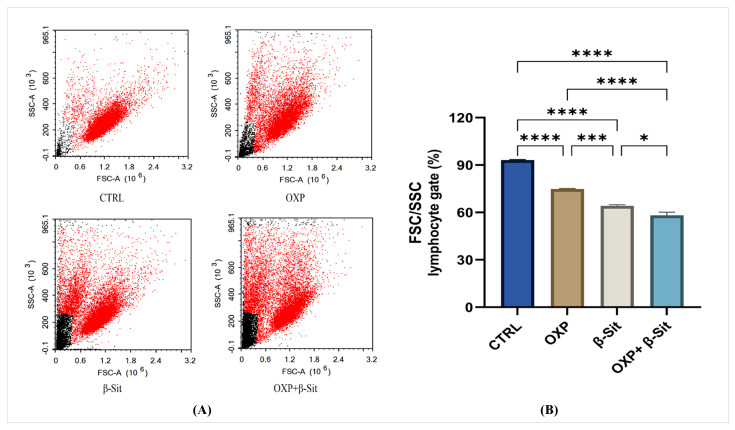
Flow cytometric analysis of FSC/SSC in COLO-205 cells after treatment with OXP, β-Sit, and OXP + β-Sit: (**A**) Histograms depicting variations in cell population, (**B**) Quantitative analysis of FSC/SSC. Data are presented as mean ± SEM (*n* = 3). Data analysis was performed using one-way ANOVA and Tukey’s post hoc test. **** *p* < 0.0001, *** *p* < 0.001, * *p* < 0.05.

The flow cytometric evaluation of FSC/SSC ratios provides further insights into the morphological changes induced by OXP, β-Sit, and the combination of both (Figure 4A,B). In CTRL cells, the majority of the population was restricted to the lymphocyte gate, indicating the normal size and low granularity characteristic of intact COLO-205 cells. The OXP treatment results in a reduction in the gated population, correlating with a loss of normal cell shape and an appearance of smaller, more granular cells experiencing stress and apoptosis. β-Sit resulted in a more significant reduction, demonstrating considerable modifications in cell structure and cytoplasmic complexity, likely indicative of necrotic and apoptotic changes. The combination treatment resulted in the lowest percentage of cells within the lymphocyte gate, highlighting the synergistic effect of OXP and β-Sit in disrupting cellular morphology.

### 2.5. Apoptosis Evaluation

**Figure 5:**
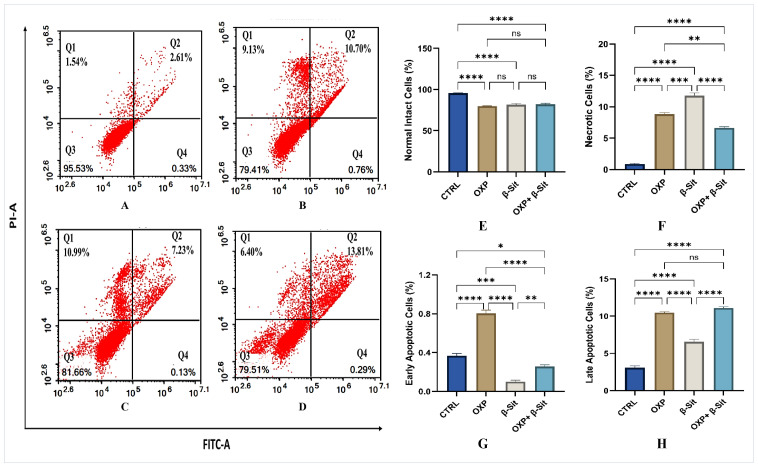
Annexin V–FITC/PI flow cytometric analysis of apoptosis in COLO-205 cells following different treatments: (**A**–**D**) Representative FITC-A/PI-A: (**A**) CTR, (**B**) OXP, (**C**) β-Sit, and (**D**) OXP + β-Sit treatments. The four quadrants represent: Q1 (necrotic cells), Q2 (late apoptotic cells), Q3 (normal intact cells), and Q4 (early apoptotic cells). (**E**–**H**) Quantitative evaluation of apoptosis: (**E**) normal intact cells, (**F**) necrotic cells, (**G**) early apoptotic cells, and (**H**) late apoptotic cells. Data are presented as mean ± SEM (*n* = 3). Statistical analysis was examined utilizing one-way ANOVA, succeeded by Tukey’s post hoc test. The significance levels are denoted as follows: **** *p* < 0.0001, *** *p* < 0.001, ** *p* < 0.01, and * *p* < 0.05. ns = not significant.

Flow cytometry and Annexin V/FITC were employed to ascertain the proportion of deceased cells [24]. Four populations were examined: normal intact, necrotic, early apoptotic, and late apoptotic cells, as depicted in Figure 5A–D. The quantitative evaluation of cell percentages via flow cytometry (Figure 5E–H) indicated that CTRL COLO-205 cells exhibited high viability with minimal apoptosis or necrosis. The OXP treatment substantially diminished the proportion of intact cells and considerably elevated early apoptosis, late apoptosis, and necrosis compared to the CTRL. β-Sit reduced cell viability, with a notable increase in late apoptotic cells and necrosis, while exhibiting the minimal decrease in early apoptosis relative to the CTRL, suggesting that early-stage apoptosis may not be the principal mechanism underlying β-Sit–induced cytotoxicity. Moreover, the combination of OXP and β-Sit resulted in the lowest proportion of viable cells, showing a reduced rate of early apoptosis along with a significant increase in late apoptosis; however, necrosis levels were lower than those observed with either β-Sit or OXP alone. These results demonstrate that the combined therapy has an effective cytotoxic impact that promotes apoptotic cell death rather than necrosis. Overall, the data demonstrate that β-Sit augments OXP-induced apoptosis and shifts the mode of cell death from necrosis toward programmed apoptosis, contributing to its enhanced anticancer efficacy.

### 2.6. Modulation of BAX, BCL2, and BAX/BCL2 Ratio by Treatments

**Figure 6:**
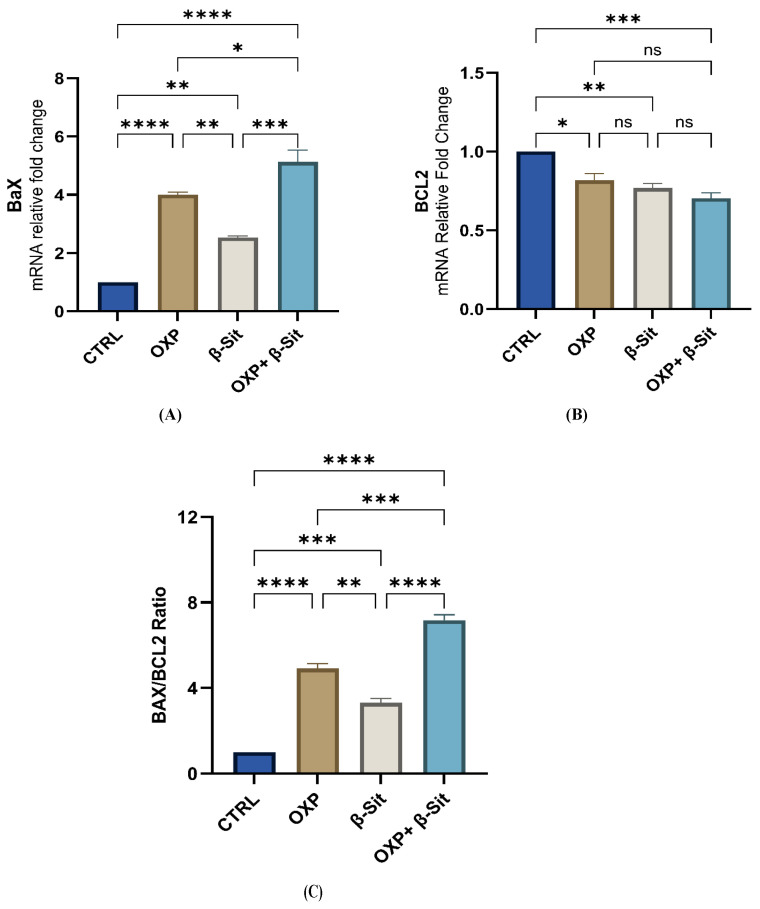
Effect of OXP, β-Sit, and their combination on (**A**) *BAX*, (**B**) *BCL2*, and (**C**) *BAX/BCL2* ratio in COLO-205 cells. Data are presented as mean ± SEM (*n* = 3). Statistical analysis was performed by one-way ANOVA and then Tukey’s post hoc test. ns = not significant, * p < 0.05, ** *p* < 0.01, *** *p* < 0.001, **** *p* < 0.0001.

The dysregulation of apoptosis is an important hallmark of cancer, and agents that induce apoptosis in cancer cells may serve as effective anticancer therapy [25]. Apoptosis, a genetically regulated mechanism of cell death, enables the orderly removal of cells to maintain homeostasis and normal development [26]. Cell survival and death are governed by the balance of BCL2 family proteins. The BCL-2 family is categorized into two groups: pro-apoptotic proteins (BAX) and pro-survival proteins (BCL-2) [27]. Moreover, upregulation of BCL2 expression serves as an impediment to apoptosis in several solid cancers [28], whereas a previous clinical study demonstrated that increased Bax levels prolonged median disease-free survival and improved chemotherapy responsiveness in ovarian cancer patients [29]. In our investigation, OXP therapy resulted in a higher increase in *BAX* expression (4-fold, *p* < 0.0001), while *BCL2* expression was considerably diminished (0.82-fold, *p* = 0.0145) relative to the CTRL. Treatment with β-Sit exhibited a comparable pattern, with *BAX* increasing by 2.53-fold (*p* = 0.0040) and *BCL2* decreasing by 0.77-fold (*p* = 0.0036) compared to CTRL. The combined treatment (OXP + β-Sit) produced the most significant effect, with *BAX* expression increasing to 5.13-fold (*p* < 0.0001) and *BCL2* expression markedly lowering to 0.70-fold compared to CTRL (*p* = 0.0007) (Figure 6A,B). Moreover, the combined treatment significantly increased *BAX* expression compared to OXP (*p* = 0.0224) or β-Sit (*p* = 0.0001) applied separately.

The *Bax/Bcl2* ratio functions as an indicator of cellular susceptibility to apoptosis [30]. Our results consistently demonstrated a significant elevation in the BAX/BCL2 ratio across all treatment groups. The *BAX/BCL2* ratio increased 4.92-fold (391.9%, *p* < 0.0001) in OXP treatment and 3.31-fold (230.7%, *p* = 0.0001) in β-Sit, relative to the CTRL. The OXP + β-Sit combination yielded the most significant effect, increasing the ratio to 7.17-fold (617.1%, *p* < 0.0001%) relative to the CTRL, signifying a substantial shift towards apoptosis. Furthermore, the OXP + β-Sit combination exhibited a significantly enhanced upregulation of the *BAX/BCL2* ratio expression relative to OXP (*p* = 0.0002) and β-Sit (*p* = <0.0001) (Figure 6C); these findings suggest that the combined treatment produced the most pronounced shift towards apoptosis compared to the individual treatments, thereby underscoring its potential efficacy.

Prior studies have indicated that β-Sit can induce apoptosis in cancer cell lines via modifying the BCL2 family, hence enhancing the observed apoptotic effect of the combination treatment. A previous work by Gu et al. [31] indicated that β-Sit markedly diminishes the proliferation and migration of HC116 cells while enhancing apoptosis. Choi et al. [23] demonstrated that β-Sit induced apoptosis in HT116 cells, correlating with a reduction in the expression of the BCL2 protein and mRNA, and an elevation in the Bax protein and mRNA. Likewise, β-Sit may prevent the proliferation of pancreatic cancer via regulating the expression of BCL2 and Bax proteins [32]. Our in silico analyses consistently revealed that β-Sit compounds possess a notable binding affinity (−6.88 kcal/mol) for BCL2, indicating their potential to inhibit BCL2 activity.

### 2.7. Impact of OXP, β-Sit, and Their Combination on the VEGF-A and PI3K/mTOR Pathways

**Figure 7:**
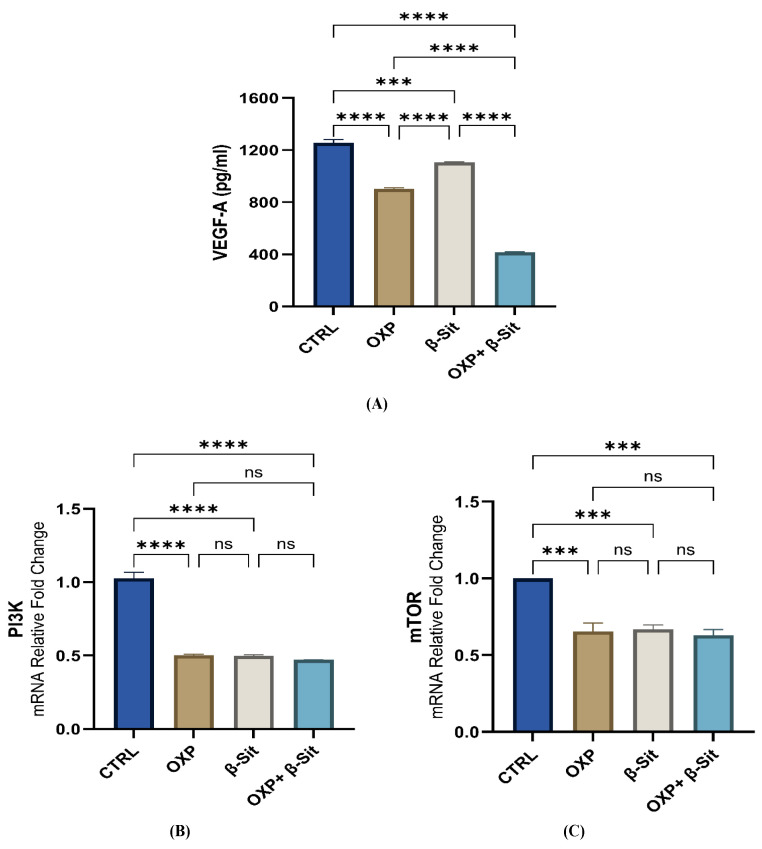
Impact of OXP, β-Sit, and OXP + β-Sit on (**A**) VEGF-A levels (pg/mL), (**B**) *PI3K* gene expression, and (**C**) *mTOR* gene expression. Data are presented as mean ± SEM (*n* = 3). Statistical analysis was via one-way ANOVA with Tukey’s post hoc test. **** *p* < 0.0001, *** *p* < 0.001, ns = not significant.

VEGF-A plays a crucial role in enhancing tumor angiogenesis, which is essential for tumor development and progression. VEGF-A, the most significant and most active pro-angiogenic factor within the VEGF family, is crucial in tumor growth. Elevated levels of VEGF-A are confirmed to correlate with unfavorable prognosis in malignancies [33], such as colorectal cancer [34,35]. Moreover, previous studies indicated that the overexpression of VEGFA markedly increased cell proliferation, migration, and invasion in vitro [36,37]. In our study, the VEGF-A levels in all treatment groups markedly diminished in comparison to the CTRL. Treatment with OXP alone resulted in a significant reduction of 28.19% (*p* < 0.0001), while β-Sit alone caused a significant decrease of 11.93% (*p* = 0.0001), and the combination treatment of OXP and β-Sit achieved the highest reduction of 66.90% (*p* < 0.0001) compared to the CTRL. Furthermore, combination therapy exhibited statistically significant improvements compared to treatment with OXP or β-Sit alone (*p* < 0.0001) (Figure 7A), suggesting a synergistic effect in angiogenesis targeting. Consistently, our in silico investigation indicates that β-Sit compounds demonstrate a promising binding affinity (−7.45 kcal/mol) to VEGFR-2, implying their potential inhibition of the VEGF signaling and hence modulating VEGF-mediated pathways, contributing to disease management [38].

The PI3K/mTOR pathway is involved in essential cellular activities, including growth, proliferation, metabolism, and survival [39]. mTOR has been linked to cell proliferation, cell cycle progress, apoptosis, necrosis, and drug resistance. [40,41,42]. Furthermore, it serves a vital role in certain malignant neoplasms [43,44]. Our results revealed that OXP, β-Sit, and their combination lowered the expression of *PI3K/mTOR* compared with the CTRL group. Furthermore, the combination therapy (OXP + β-Sit) exhibited the most pronounced decrease in *PI3K/mTOR* expression relative to other treatments; nevertheless, this reduction was not statistically significant (Figure 7B,C), suggesting a trend toward enhanced inhibition of *PI3K/mTOR* expression. The pro-apoptotic impact of β-Sit is partially attributed to the inhibition of the PI3K/mTOR signaling pathway. This effect is evidenced by β-Sit’s ability to diminish AKT/mTOR phosphorylation across several cancer cell lines, while the PI3K inhibitor markedly enhanced β-Sit-induced cytotoxicity [45,46,47]. Consistently, our in silico docking study indicates that β-Sit interacts with PI3K (binding affinity: −8.02 kcal/mol), suggesting a potential role in its cytotoxic action.

### 2.8. Impact of OXP, β-Sit, and Their Combination on β-Catenin and NF-κB-p65 Protein Expression

**Figure 8:**
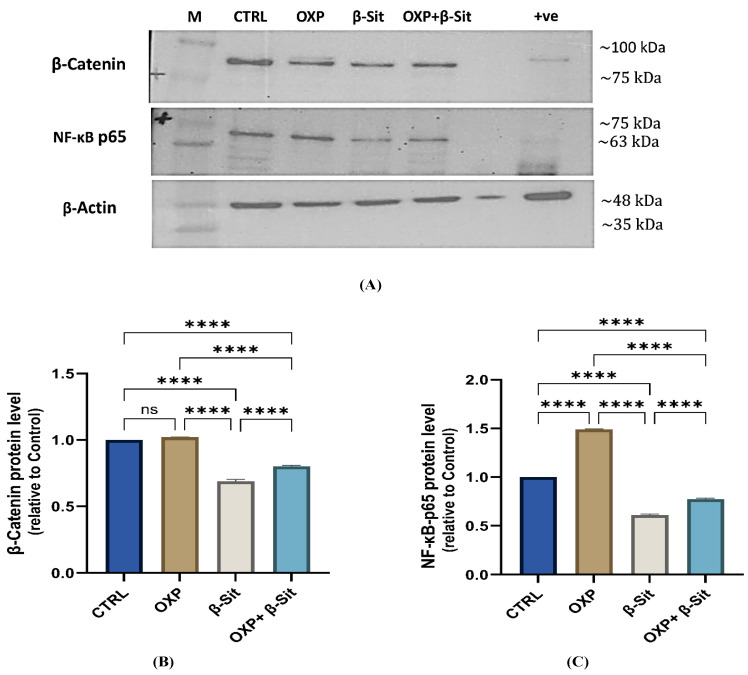
Impact of treatment on β-Catenin and NF-κB-p65 in COLO-205 Cells: (**A**) Western blot analysis of β-Catenin, NF-κB-p65, and β-actin protein, (**B**) β-Catenin protein level, and (**C**) NF-κB-p65 protein level. Data are presented as mean ± SEM (*n* = 3). Statistical analysis was conducted by one-way ANOVA/Tukey’s post hoc test. **** *p* < 0.0001, ns = not significant.

The Western blot results in Figure 8A,B demonstrated that OXP elevated β-catenin expression with no statistical significance (~1.02-fold, *p* = 0.273), while β-Sit significantly reduced β-catenin levels (~0.69-fold, *p* < 0.0001), and the combined treatment resulted in moderate suppression (~0.80-fold, *p* < 0.0001) relative to CTRL. Moreover, OXP treatment increased NF-κB-p65 expression by 1.49-fold (*p* < 0.0001 compared to CTRL), signifying the activation of a survival mechanism. In contrast, β-Sit markedly reduced NF-κB-p65 levels (0.61-fold, *p* < 0.0001), while the combination of OXP and β-Sit resulted in intermediate expression (0.77-fold, *p* < 0.0001) relative to CTRL. Furthermore, β-Sit, either alone or combined with OXP, substantially diminished the levels of β-catenin and NF-κB-p65 in comparison to OXP alone (*p* < 0.0001), indicating that β-Sit mitigated the OXP-induced increase of β-catenin and NF-κB-p65. This aligns with prior studies indicating that β-Sit can downregulate β-catenin expression [48,49] and promote apoptosis in CC cells by targeting β-catenin [50]. Moreover, β-Sit inhibited the NF-κB pathway, which often allows tumor cells to evade apoptosis by upregulating pro-survival genes [32]. Furthermore, Shen et al. [51] revealed that β-Sit therapy concurrently inhibits NF-κB-p65 and β-catenin signaling. The data suggest that β-Sit may enhance OXP efficacy through improving the downregulation of Wnt/β-catenin signaling and suppressing NF-κB-mediated resistance pathways.

### 2.9. Effect of Treatment on Interferon Gamma (IFN-γ) and Total Antioxidant Capacity (TAC)

**Figure 9:**
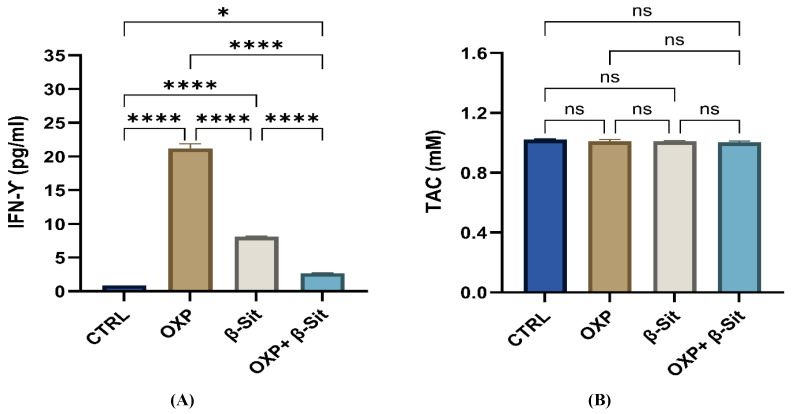
Effects of OXP, β-Sit, and OXP + β-Sit on (**A**) IFN-γ level (pg/mL) and (**B**) TAC concentration (mM). Data are presented as mean ± SEM (*n* = 3). Statistical analysis was performed using one-way ANOVA/Tukey’s post hoc test. * *p* < 0.05, **** *p* < 0.0001, ns = not significant.

IFN-γ exhibits multifaceted anti-tumor functions in cancer cells, involving anti-proliferative, pro-apoptotic, and anti-angiogenic processes [52,53]. Our data indicated that OXP therapy significantly increased the IFN-γ level, corroborating the findings of Hu et al. [54], who also observed that OXP increases anti-tumor immunity by increasing IFN-γ production. β-Sit also resulted in a moderate elevation of IFN-γ, indicating its established immunomodulatory effect [32,51]. In the combination treatments, IFN-γ levels were lower than in each individual treatment (Figure 9A). This decrease may be attributed to enhanced cell death (elevated Sub-G1 population), which limits the percentage of viable cells able to secrete cytokines, or a shift from immunological signaling effects to direct apoptotic mechanisms, as further corroborated by the observed alterations in BAX/BCL2 expression. On the other hand, the TAC values demonstrate no variation among groups. This outcome suggests that the principal mechanism of action for OXP, β-Sit, and their combination does not involve alterations in overall antioxidant capacity, but rather the modulation of apoptotic and survival signaling pathways (Figure 9B).

### 2.10. Study Limitations and Future Perspectives

This study was limited to in vitro investigations that used only one CC cell line (COLO-205). Furthermore, normal colon cells were not included to evaluate selective cytotoxicity. Future research will aim to assess the in vivo effects of β-Sit and OXP, investigate their molecular mechanisms, and incorporate non-tumorigenic colon cells to evaluate selective cytotoxicity and therapeutic safety.

## 3. Materials and Methods

### 3.1. Materials

COLO-205 cells were procured from Nawah Scientific Inc. (Cairo, Egypt). OXP was purchased from Mylan SAS (Saint-Priest, France), and β-Sit was acquired from Source Naturals (Scotts Valley, CA, USA). The β-Catenin (Cat. No. SC-7963) and NF-κB-p65 (Cat. No. SC-8008) antibodies (Ab) were procured from Santa Cruz Biotechnology, Inc. (Dallas, TX, USA). β-Actin (Cat. No. MA5-11869) and secondary Ab rabbit anti-mouse IgG (Cat. No. 61-6520), HRP, were obtained from Invitrogen (Carlsbad, CA, USA). The BLUelf prestained protein ladder (Cat. No. PM008-0500) was purchased from GeneDireX, Inc. (Taoyuan City, Taiwan). All residual compounds were of analytical grade.

### 3.2. Molecular Docking Analysis

Molecular docking was performed to evaluate the potential affinity of β-Sit for target proteins: BCL2, PI3K, and VEGFR-2 (PDB codes: 6gl8, 5xgi, and 3b8r) [55,56,57]. At first, water molecules and undesired molecules were removed from the protein complexes. Crystallographic protons were then incorporated, and missing valence atoms were adjusted. The protein structure was energy-minimized and saved as a PDBQT file. The two-dimensional (2D) structure of β-Sit was created using Chem-Bio Draw Ultra 16.0, saved as an SDF file, and then transformed into a 3D. Protonation and energy minimization were performed and saved as a PDBQT file; the generated ligands were evaluated against the prior targets. The top-scoring poses were selected for each target protein, and the procedures were executed utilizing Autodock Vina 1.5.7 software. Docking was conducted using a rigid receptor approach, in which the protein remained fixed while β-Sit was allowed flexibility. During refinement, the ligand generated twenty distinct conformations. During the docking refinement, β-Sit was permitted to produce twenty distinct positions. The docking procedure was confirmed through the redocking of the co-crystallized ligands for each protein, with RMSD values remaining below 2 Å, thereby validating the docking procedure. Furthermore, the docking scores of the most optimal postures with the active sites were documented, and 3D and 2D representations were created with the Discovery Studio 2024 visualizer [58].

### 3.3. Cell Culture

The cells were cultured in RPMI media enriched with 100 mg/mL streptomycin, 10% heat-inactivated fetal bovine serum, and 100 U/mL penicillin in a humidified atmosphere with 5% CO_2_ at 37 °C. The COLO-205 cell line was chosen due to its prevalent application in colorectal cancer research for examining drug-induced apoptosis and associated signaling pathways [59]. Furthermore, in our investigation, the doses of OXP, β-Sit, or the combination therapy were identified based on preliminary MTT assays to estimate the relevant IC_50_ values.

### 3.4. Evaluation of Cytotoxicity

Cell viability was assessed using the MTT method. 100 μL of cell suspension (5 × 10^3^ cells) was inoculated onto 96-well plates and cultured in complete medium for 24 h at 37 °C. Subsequently, the cells were treated with 100 μL of media enriched with OXP, β-Sit (dissolved in dimethyl sulfoxide (DMSO) [60]), and OXP + β-Sit at various concentrations (0.1, 0.3, 1, 3, 10, 30, 100, 300, and 500 μM). Furthermore, a vehicle control (cells treated only with DMSO, without β-Sit) was included. Following 48 h of compound exposure, the medium was discarded, and 20 μL of MTT solution (1 mg/mL stock) was introduced to each well containing 100 μL of phosphate-buffered saline (PBS), then incubated at 37 °C for 4 h. The resulting formazan crystals were dissolved with 100 μL of absolute DMSO. The absorbance of the dissolved formazan was quantified at λmax 570 nm utilizing a multi-well plate reader (BMG LABTECH^®^ FLUOstar Omega, Ortenberg, Germany).

### 3.5. Cell Cycle Analysis

Following treatment at IC50 with OXP at 25.64 µM, β-Sit at 275.9 µM, and a combination of OXP (3.24 µM) and β-Sit (36.01 µM) for 48 h, 1 × 10^5^ cells were collected by trypsinization and washed with ice-cold PBS (pH 7.4). The cells were then fixed by suspension in 2 mL of ice-cold 60% ethanol, incubated at 4 °C for 1 h, washed with PBS, and then resuspended in 1 mL of PBS containing RNase A (50 μg/mL) and propidium iodide (PI, 10 μg/mL). After 20 min incubation in darkness at 37 °C, the cell DNA content was assessed via flow cytometry utilizing the FL2 (λex/em = 535/617 nm) channel on an ACEA NovoCyte™ flow cytometer (ACEA Biosciences Inc., San Diego, CA, USA). A total of 12,000 events were documented for each sample, and cell cycle distribution was evaluated utilizing ACEA NovoExpress™ software (version 1.1.0, ACEA Biosciences Inc., San Diego, CA, USA).

### 3.6. Analysis of Apoptosis

Apoptosis analysis was conducted with an Annexin V-FITC apoptosis detection kit (Abcam Inc., Cambridge Science Park, Cambridge, UK) combined with dual fluorescence channel flow cytometry. Following a 48 h treatment with OXP, β-Sit, and OXP + β-Sit, 1 × 10^5^ cells were collected using trypsinization and washed with ice-cold PBS. The cells were then treated with 0.5 mL of Annexin V-FITC/PI solution and incubated for 30 min in the dark at room temperature. Thereafter, the cells were evaluated using an ACEA Novocyte™ flow cytometer (ACEA Biosciences Inc., San Diego, CA, USA) and analyzed for FITC and PI fluorescent signals with the FL1 and FL2 signal detectors, respectively (λex/em 488/530 nm for FITC and λex/em 535/617 nm for PI). Data from 12,000 events were collected for each sample, and cells positive for FITC and PI were quantified by quadrant analysis utilizing ACEA NovoExpress™ software (ACEA Biosciences Inc., San Diego, CA, USA).

### 3.7. Quantitative RT-PCR Analysis

The total RNA was extracted from the samples by a TransZol Up Plus RNA Kit (Cat. ER501, TransGen Biotech Co., Ltd., Beijing, China) in accordance with the manufacturer’s instructions. The content and quality of RNA were assessed with a FLUOstar Omega Plate Reader (BMG LABTECH) spectrophotometer in accordance with the A260/280 ratio. Then, the RNA was reverse transcribed into cDNA using the EasyScript First-Strand cDNA Synthesis SuperMix Kit (Cat. AE301, TransGen Biotech Co., Ltd., Beijing, China) following the manufacturer’s guidelines. Gene expression was assessed by PerfectStart Green qPCR SuperMix (Cat. No. AQ601, TransGen Biotech, China) on the Bio-Rad platform (Bio-Rad CFX OPUS 96; Bio-Rad, Hercules, CA, USA). Relative gene expression was assessed by the 2^−ΔΔCt^ method. The primer sequence of all studied genes was provided in Table 2.

### 3.8. Colorimetric and Enzyme-Linked Immunosorbent Assay (ELISA) Analysis

TAC was measured using a colorimetric method with a commercial kit (Biodiagnostic, Giza, Egypt); levels of VEGF-A and IFN-γ were quantified using commercially available ELISA kits (Cat. Nos. E-EL-H0111 and E-EL-H0108; Elabscience Biotechnology Inc., Houston, TX, USA), in accordance with the manufacturer’s instructions.

### 3.9. Western Blotting Analysis

Tissues were lysed in RIPA buffer containing a protease and phosphatase inhibitor cocktail, then agitated for 30 min at 4 °C, and centrifuged at 16,000× *g* for 20 min at 4 °C. The supernatants were collected and kept on ice. Protein was quantified with the bicinchoninic acid method. The total protein from each sample was mixed with 2× Laemmli buffer, heated at 95 °C for 5 min, and subsequently centrifuged briefly at 16,000× *g*. Proteins were loaded into the wells of a 15% mini SDS-PAGE gel, accompanied by a molecular weight marker. The gels were electrophoresed for 5 min at 90 V for the stacking gel and then at 100–150 V for the separating gel. Gels were equilibrated in 1× transfer buffer for 10–15 min after electrophoresis, and then proteins were transferred to 0.2 µm PVDF membranes (Sigma-Aldrich, St. Louis, MO, USA). The cassette was placed in the transfer tank holding an ice block to prevent overheating. The transfer operation was conducted at 100 V for a duration of 30 min to 2 h. The membrane was blocked with 5% skimmed milk (SM) in TBST for 1 h, followed by incubation with a primary antibody diluted in 5% BSA in TBST at 4 °C. The membrane was washed with TBST, incubated with HRP-conjugated secondary antibody diluted in SM in TBST for 1 h at room temperature, then washed again. Bands were detected using an enhanced chemiluminescent (ECL) substrate (Thermo Fisher Scientific, Waltham, MA, USA). Chemiluminescence was identified via the Biorad System, and the band intensities of the target proteins were quantified by image analysis software.

### 3.10. Statistical Analysis

Data analysis was conducted by GraphPad Prism Software (version 10.4.2, GraphPad Software Inc., San Diego, CA, USA). IC_50_ values were determined by nonlinear regression analysis by fitting the experimental data to a log(inhibitor) vs. response (three-parameter logistic) model. The quality of the curve fitting was assessed using the coefficient of determination (R^2^), which ranged between 0.81 and 0.97 for all treatments, confirming a satisfactory goodness of fit. The data are shown as the mean ± SEM (*n* = 3). Statistical significance among treatment groups was assessed using one-way ANOVA followed by Tukey’s post hoc test, with *p* < 0.05 considered statistically significant.

## 4. Conclusions

In conclusion, our research indicates that the combination of OXP and β-Sit resulted in a reduction in IC_50_, which was achieved by 3.24 µM of OXP and 36.01 µM of β-Sit, highlighting the strong synergistic effect. β-Sit augments the anticancer potency of OXP by promoting apoptosis, elevating the BAX/BCL2 ratio, and increasing the sub-G1 population. Furthermore, the combination of OXP and β-Sit enhances the inhibitory effect on angiogenesis by diminishing VEGF-A levels and inhibiting β-catenin and NF-κB-p65, thereby preventing the proliferation and viability of resistant cancer cells. These findings offer initial insights that require further investigation through extensive in vitro and in vivo studies.

## Figures and Tables

**Table 1:** Molecular docking of β-Sit against VEGFR-2, PI3K, and BCL2.

Tested Compounds	Compounds	RMSD Value (Å)	Docking (Affinity) Score (kcal/mol)
VEGFR-2	β-Sit	1.41	−7.45
	Co-crystalized ligand (887)	0.79	8.13
PI3K	β-Sit	1.26	−7.98
	Co-crystalized ligand (84R)	0.85	−8.02
BCL2	β-Sit	1.85	−6.88
	Co-crystalized ligand (F3Q)	1.44	7.11

Vascular Endothelial Growth Factor Receptor-2 (VEGFR-2), Phosphoinositide 3-Kinase (PI3K), B-Cell Lymphoma 2 (Bcl-2).

**Table 2:** Sequence of primers for all analyzed genes.

	Forward Sequence	Reverse Sequence	Genes Accession Numbers
*GAPDH*	GTC TCC TCT GAC TTC AAC AGC G	ACC ACC CTG TTG CTG TAG CCA A	NM_002046
*Bcl2*	ATC GCC CTG TGG ATG ACT GAG T	GCC AGG AGA AAT CAA ACA GAG GC	NM_000633
*Bax*	TCA GGA TGC GTC CAC CAA GAA G	TGT GTC CAC GGC GGC AAT CAT C	NM_138761
*mTOR*	AGC ATC GGA TGC TTA GGA GTG G	CAG CCA ATC TTT GGA GAC C	NM_004958
*PI3K*	GAA GCA CCT GAA TAG GCA AGT CG	GAG CAST CCA TGA AAT CTG GTC GC	NM_006218

Glyceraldehyde-3-phosphate dehydrogenase (*GAPDH*), B-cell lymphoma 2 (*BCL2*); Bcl-2–associated X protein (*BAX*); phosphoinositide 3-kinase (*PI3K*); mechanistic target of rapamycin (*mTOR*).

## Data Availability

The original contributions presented in this study are included in the article. Further inquiries can be directed to the corresponding author.
